# Supplementary material for: Nucleoredoxin regulates WNT signaling during pituitary stem cell differentiation
Source: bioRxiv. 2025 Feb 3:2025.01.30.635771. Preprint. [Version 2] doi: 10.1101/2025.01.30.635771 (PMC11838423; doi:10.1101/2025.01.30.635771)
Supplement: Supplement 1 [file media-1.pdf]

Supplementary Material

Supplementary Table 1. Antibody sources, dilutions and detection methods

Target Protein	Primary Antibody	Source	Dilution	Antigen Retrieval	Blocking	Secondary Antibody	Tertiary reagent	Detection
GH	Monkey anti-GH	National Hormone & Peptide Program, #AFP411S	1:1000	none	2%BSA, 1% normal goat serum, in 1xPBS	Goat anti-human Alexa Flour 488	none	
TSHB	Guinea pig anti-TSHb	National Hormone & Peptide Program, #AFP967793	1:500	none	2%BSA, 1% normal goat serum, in 1xPBS	Goat anti-guinea pig Alexa Fluor 488	none	
POMC	Guinea pig anti-ACTH	National Hormone & Peptide Program, #AFP71111591	1:500	none	2%BSA, 1% normal goat serum, in 1xPBS	Goat anti-guinea pig Alexa Fluor 488	none	
PECAM1	Rabbit anti-PECAM	Thermo Scientific #RB-10333	1:100	10 min boiling 0.1M Citric Acid, pH 6.0	2%BSA, 1% normal donkey serum, in 1xPBS	Donkey anti-rabbit Biotin	Strep-HRP	TSA CF488, Biotium #33002
POU1F1	Rabbit anti-POU1F1	Dr. Simon Rhodes	**1:500**	10 min boiling 0.1M Citric Acid, pH 6.0	2%BSA, 1% normal donkey serum, in 1xPBS	Donkey anti-rabbit Biotin	Strep-HRP	TSA CF488, Biotium #33002
LEF1	Goat anti-LEF1	Santa Cruz Biotechnology	1:500	10 min boiling 0.1M Citric Acid, pH 6.0	2%BSA, 1% normal donkey serum, in 1xPBS	Donkey anti-goat Biotin	Strep-HRP	TSA Alexa Fluor 488, Life Tecnologies#T20932
LHB	Guinea pig anti-LHB	National Hormone & Peptide Program	1:50	None	2%BSA, 1% normal donkey serum, in 1xPBS	Goat anti-guinea pig Alex Fluor 488	none	
NR5A1	Rabbit anti-NR5A1	custom	1:1000	10mM Citric Acid, pH 6.0	2%BSA, 1% normal donkey serum, in 1xPBS	Donkey anti-rabbit Biotin	Strep-HRP	TSA CF488, Biotium #33002

Supplemental Table 2. Mendelian distribution of *Nxn* genotypes during development

genotype	age[1]			
	e10.5-e11.5	e14.5	e17.5-e18.5	2-3 wk
+/+	13 (17)	11 (26)	23 (28)	30 (29)
+/-	47 (62)	21 (49)	33 (40)	86 (58)
-/-	16 (21)	11 (26)	26 (32)	0 (29)
p value	0.11	0.99	0.19	0.001**

[1]number of fetuses (percentage of total)

Supplemental Table 3. Phenotype information for homozygous and heterozygous *Nxn* alleles in mice. Multiple *Nxn* alleles have been generated and phenotyped by the International Mouse Phenotyping Consortium (IMPC) (Supplemental Fig. 1) [73]. Phenotypes for these alleles are directly deposited with Mouse Genome Informatics (MGI) (www.informatics.jax.org/) and are available for download from the IMPC website (www.mousephenotype.org).

feature	allele	genotype	gender	age	method of assessment	parameter	institution	most significant p value
abnormal mammary gland morphology	Nxn<tm1b(EUCOMM)Wtsi>	heterozygote	male	Early adult	Gross Pathology and Tissue Collection	Mammary gland	CCP-IMG	0
hemorrhage	Nxn<em1(IMPC)Mbp>	heterozygote	female	E15.5	Gross Morphology Embryo E14.5-E15.5	Hemorrhage	UC Davis	0
unresponsive to tactile stimuli	Nxn<em1(IMPC)Bay>	heterozygote	male	E18.5	Gross Morphology Embryo E18.5	Responsive to tactile stimuli	BCM	0
decreased cardiac muscle contractility	Nxn<tm1a(EUCOMM)Wtsi>	heterozygote	female	Early adult	Echo	Ejection Fraction	BCM	0.00000442505718657911
small adrenal glands	Nxn<tm1b(EUCOMM)Wtsi>	heterozygote	male	Early adult	Gross Pathology and Tissue Collection	Adrenal gland	TCP	0
decreased body length	Nxn<em1(IMPC)Mbp>	heterozygote	female	Early adult	Organ Weight	Body length	UC Davis	0.0000625759941515103
increased fasting circulating glucose level	Nxn<em1(IMPC)J>	heterozygote	not_considered	Early adult	Intraperitoneal glucose tolerance test (IPGTT)	Fasted blood glucose concentration	JAX	0.00000774424608485091
increased circulating bilirubin level	Nxn<em1(IMPC)J>	heterozygote	male	Early adult	Clinical Chemistry	Total bilirubin	JAX	0.00000831715513188009
decreased locomotor activity	Nxn<tm1b(EUCOMM)Wtsi>	heterozygote	female	Early adult	Open Field	Periphery resting time	ICS	3.78027599410494e-11
unresponsive to tactile stimuli	Nxn<tm1b(EUCOMM)Wtsi>	heterozygote	female	E18.5	Gross Morphology Embryo E18.5	Responsive to tactile stimuli	BCM	0
spina bifida	Nxn<em1(IMPC)Mbp>	heterozygote	male	E15.5	Gross Morphology Embryo E14.5-E15.5	spina bifida	UC Davis	0
abnormal behavior	Nxn<tm1a(EUCOMM)Wtsi>	heterozygote	male	Early adult	Open-field	Latency to centre entry	HMGU	0.00006443007269329
irregularly shaped pupil	Nxn<tm1a(EUCOMM)Wtsi>	heterozygote	female	Early adult	Eye Morphology	Pupil Shape	BCM	0.0000189450375385945
abnormal digit morphology	Nxn<tm1b(EUCOMM)Wtsi>	heterozygote	male	Early adult	X-ray	Digit integrity	ICS	7.98216167332279e-7
abnormal optic disk morphology	Nxn<tm1b(EUCOMM)Wtsi>	heterozygote	not_considered	Early adult	Eye Morphology	Optic Disc	MARC	0.0000135826410211897
abnormal cranium morphology	Nxn<tm1b(EUCOMM)Wtsi>	heterozygote	female	Early adult	X-ray	Skull shape	WTSI	0.0000240980780299203
abnormal locomotor behavior	Nxn<tm1b(EUCOMM)Wtsi>	heterozygote	female	Early adult	Fear Conditioning	% Freezing duration habituation-1	ICS	0.0000667798700178943
decreased exploration in new environment	Nxn<tm1b(EUCOMM)Wtsi>	heterozygote	female	Early adult	Open Field	Latency to center entry	ICS	8.73716271937624e-8
abnormal snout morphology	Nxn<tm1b(EUCOMM)Wtsi>	heterozygote	female	Early adult	Combined SHIRPA and Dysmorphology	Snout size	MRC Harwell	0.00000354288659733035
anophthalmia	Nxn<tm1a(EUCOMM)Wtsi>	heterozygote	female	Early adult	Gross Pathology and Tissue Collection	Eye with optic nerve	BCM	0
increased grip strength	Nxn<tm1a(EUCOMM)Wtsi>	heterozygote	female	Early adult	Grip-Strength	Forelimb grip strength measurement mean	HMGU	0.000376182682050536
abnormal freezing behavior	Nxn<tm1b(EUCOMM)Wtsi>	heterozygote	female	Early adult	Fear Conditioning	% Freezing duration pre-cue-1	ICS	0.0000128431465095489
abnormal eyelid morphology	Nxn<tm1b(EUCOMM)Wtsi>	heterozygote	female	Early adult	Eye Morphology	Eyelid morphology	WTSI	0.000017813517915287
increased effector memory CD8-positive, alpha-beta T cell number	Nxn<tm1b(EUCOMM)Wtsi>	heterozygote	male	Early adult	Immunophenotyping	Effector CD8+ T cells - % of live leukocytes (Panel A)	CCP-IMG	1.13003262392377e-7
increased mean corpuscular hemoglobin concentration	Nxn<em1(IMPC)J>	heterozygote	female	Early adult	Hematology	Mean cell hemoglobin concentration	JAX	0.000127967928617623
increased circulating triglyceride level	Nxn<tm1b(EUCOMM)Wtsi>	heterozygote	male	Early adult	Clinical Chemistry	Triglycerides	CCP-IMG	0.000461664346270157
short tibia	Nxn<tm1b(EUCOMM)Wtsi>	heterozygote	male	Early adult	X-ray	Tibia length	UC Davis	0.00000120895797714198
increased circulating alkaline phosphatase level	Nxn<tm1b(EUCOMM)Wtsi>	heterozygote	female	Early adult	Clinical Chemistry	Alkaline phosphatase	ICS	0.000249869880566859
abnormal retina blood vessel morphology	Nxn<em1(IMPC)Bay>	heterozygote	male	Early adult	Eye Morphology	Retinal Blood Vessels Pattern	BCM	0.0000063102777126984
increased circulating HDL cholesterol level	Nxn<tm1b(EUCOMM)Wtsi>	heterozygote	male	Early adult	Clinical Chemistry	HDL-cholesterol	CCP-IMG	0.000320734982264727
preweaning lethality, incomplete penetrance	Nxn<tm1a(EUCOMM)Wtsi>	homozygote	male	Early adult	Viability Primary Screen	Homozygous males viability	BCM	-
abnormal head shape	Nxn<tm1b(EUCOMM)Wtsi>	homozygote	male	E18.5	Gross Morphology Embryo E18.5	Head shape	BCM	0
abnormal embryo size	Nxn<em1(IMPC)Bay>	homozygote	male	E18.5	Gross Morphology Embryo E18.5	Embryo Size	BCM	0
abnormal facial morphology	Nxn<tm1b(EUCOMM)Wtsi>	homozygote	male	E18.5	Gross Morphology Embryo E18.5	Facial Morphology	BCM	0
cleft palate	Nxn<tm1b(EUCOMM)Wtsi>	homozygote	male	E18.5	Gross Morphology Embryo E18.5	Cleft palate	BCM	0
preweaning lethality, complete penetrance	Nxn<em1(IMPC)Mbp>	homozygote	female	Early adult	Viability Primary Screen	Homozygous females viability	UC Davis	0
abnormal facial morphology	Nxn<em1(IMPC)Bay>	homozygote	male	E18.5	Gross Morphology Embryo E18.5	Facial Morphology	BCM	0
spina bifida	Nxn<em1(IMPC)Mbp>	homozygote	female	E15.5	Gross Morphology Embryo E14.5-E15.5	spina bifida	UC Davis	0
cleft palate	Nxn<em1(IMPC)J>	homozygote	female	E18.5	Gross Morphology Embryo E18.5	Cleft palate	JAX	0
preweaning lethality, complete penetrance	Nxn<tm1b(EUCOMM)Wtsi>	homozygote	male	Early adult	Viability Primary Screen	Homozygous males viability	BCM	0
facial cleft	Nxn<tm1b(EUCOMM)Wtsi>	homozygote	female	E18.5	Gross Morphology Embryo E18.5	Facial Cleft	BCM	0
unresponsive to tactile stimuli	Nxn<tm1b(EUCOMM)Wtsi>	homozygote	female	E18.5	Gross Morphology Embryo E18.5	Responsive to tactile stimuli	BCM	0
hemorrhage	Nxn<em1(IMPC)Mbp>	homozygote	male	E15.5	Gross Morphology Embryo E14.5-E15.5	Hemorrhage	UC Davis	0
preweaning lethality, complete penetrance	Nxn<em1(IMPC)Bay>	homozygote	not_considered	Early adult	Viability Primary Screen	Homozygous animals viability	BCM	0
abnormal craniofacial morphology	Nxn<tm1b(EUCOMM)Wtsi>	homozygote	female	E12.5	Gross Morphology Embryo E12.5	Craniofacial morphology	JAX	0
cleft palate	Nxn<tm1b(EUCOMM)Wtsi>	homozygote	male	E15.5	Gross Morphology Embryo E14.5-E15.5	Cleft Palate	JAX	0
abnormal head shape	Nxn<em1(IMPC)Bay>	homozygote	male	E18.5	Gross Morphology Embryo E18.5	Head shape	BCM	0
unresponsive to tactile stimuli	Nxn<em1(IMPC)Bay>	homozygote	male	E18.5	Gross Morphology Embryo E18.5	Responsive to tactile stimuli	BCM	0
abnormal embryo size	Nxn<em1(IMPC)J>	homozygote	male	E18.5	Gross Morphology Embryo E18.5	Embryo Size	JAX	0
abnormal embryo size	Nxn<tm1b(EUCOMM)Wtsi>	homozygote	female	E18.5	Gross Morphology Embryo E18.5	Embryo Size	BCM	0
abnormal head size	Nxn<tm1b(EUCOMM)Wtsi>	homozygote	female	E18.5	Gross Morphology Embryo E18.5	Head size	BCM	0
abnormal facial morphology	Nxn<em1(IMPC)J>	homozygote	male	E18.5	Gross Morphology Embryo E18.5	Facial Morphology	JAX	0
abnormal craniofacial morphology	Nxn<tm1b(EUCOMM)Wtsi>	homozygote	no data	E15.5	Gross Morphology Embryo E14.5-E15.5	Craniofacial morphology	TCP	0
abnormal embryo size	Nxn<tm1b(EUCOMM)Wtsi>	homozygote	male	E15.5	Gross Morphology Embryo E14.5-E15.5	Embryo Size	JAX	0
facial cleft	Nxn<em1(IMPC)Bay>	homozygote	male	E18.5	Gross Morphology Embryo E18.5	Facial Cleft	BCM	0
no spontaneous movement	Nxn<tm1b(EUCOMM)Wtsi>	homozygote	no data	E18.5	Gross Morphology Embryo E18.5	Spontaneous movement	CCP-IMG	0
abnormal head size	Nxn<em1(IMPC)Bay>	homozygote	male	E18.5	Gross Morphology Embryo E18.5	Head size	BCM	0
preweaning lethality, complete penetrance	Nxn<em1(IMPC)J>	homozygote	not_considered	Early adult	Viability Primary Screen	Viability Outcome	JAX	0
facial cleft	Nxn<em1(IMPC)J>	homozygote	male	E18.5	Gross Morphology Embryo E18.5	Facial Cleft	JAX	0

Supplemental Table 4. Clinical features of individuals with *NXN* variants.

Patient identifier	inheritance	DNA	Protein	Features #	Ref
1 (8841)	homozygous	Stop gain, c.625C>T	p.Arg209*	GHD, CP, Jaw, Face, Meso, Br, He, Ki and more	[51]
2 (9844)	Compound heterozygote	In frame deletion, c.1234-1236; 84 kb deletion including exon 1 of *NXN*	p.Glu412del, none	CP, Jaw, Face, Meso, Br	[51]
3 (9847)	Compound heterozygote	Same as sister above		Jaw, Face, Meso, Br	[51]
4 (10973)	Compound heterozygote	Del17p.13.3, *NXN* and 12 other genes; nonsense, c.817C>T	None, p.Gln273*	Face, Meso, Br	[9]

# Feature code:
GHD = Growth hormone deficiency
CP = Cleft palate, absent uvula
Jaw = micrognathia
Face = facial characteristics
Meso = mesomelia
Br = brachydactyly
He = heart: bicuspid aortic valve, abnormal right coronary artery
Ki = left kidney duplication
More = omphalocele, 2x ventral hernias, ear infection, conductive HL

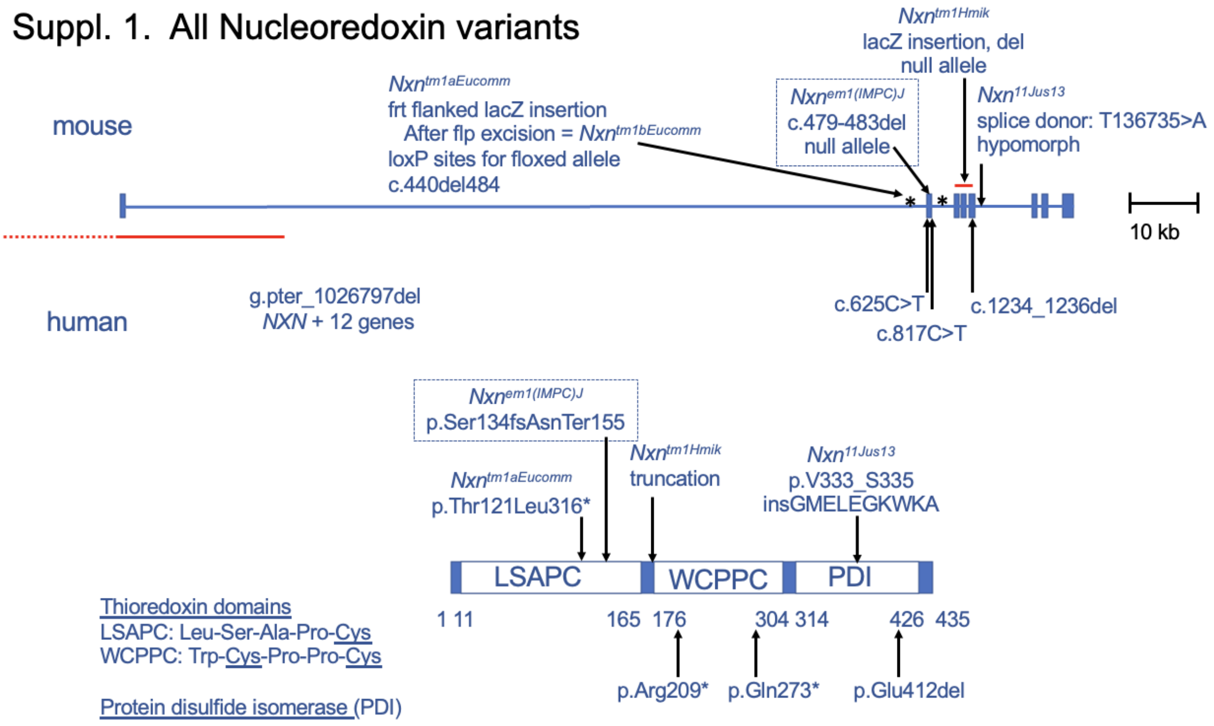

<u>Supplemental Figure 1. Nucleoredoxin variants in mouse and man.</u> The structure of the human and mouse nucleoredoxin genes are similar. Mouse alleles are indicated above the gene diagram, including the knockout first *Nxn*^{tm1aEucomm} allele [53], the *Nxn*$^{em1(IMPC)J}$ allele described in this manuscript, the lacZ insertion *Nxn*^{tm1Hmik} allele [1], and the hypomorphic *Nxn*^{11Jus13} allele [52]. Patient mutations in *NXN* are indicated below the gene diagram including an individual homozygous for stop gain, c.625C>T, two compound heterozygous sisters with an 84 kb deletion including exon 1 of *NXN* (red) and an in frame deletion, c.1234-1236del, and an unrelated compound heterozygote with deletion of *NXN* and 12 other genes in trans with a nonsense mutation, c.817C>T, as described [8,9,51]. The NXN protein consists of three domains indicated in the protein diagram. All variants in mouse and man are expected to be null alleles due to the loss of key protein domains, except the hypomorphic *Nxn*^{11Jus13} allele which splices normally at a low frequency.

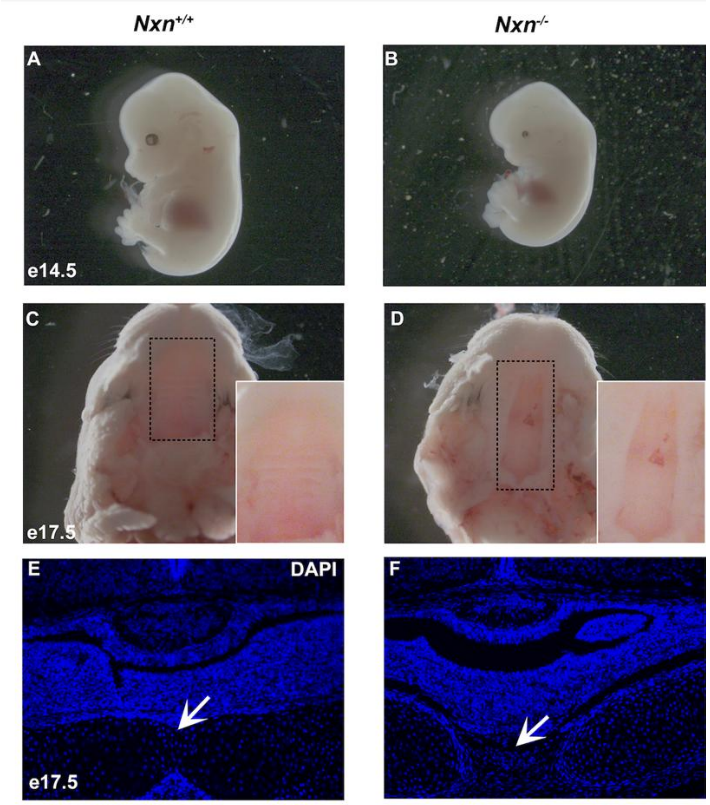

Supplemental Figure 2. Craniofacial abnormalities in *Nxn* mutants.

Wild type (A) and *Nxn* mutants (B) were collected at e14.5. None of the wild type animals had micro-ophthalmia or anophthalmia, but some of the mutants did.

Wild type (C) and mutant (D) animals were collected at e17.5, and the palates were examined. The palate area is enlarged in the inset.

Wild type (E) and mutant (F) animals were collected at e17.5, fixed, sectioned in the coronal plane, and stained with DAPI. The basisphenoid bone is fused at the base of the pituitary gland in wild type, but not mutant animals (arrows).

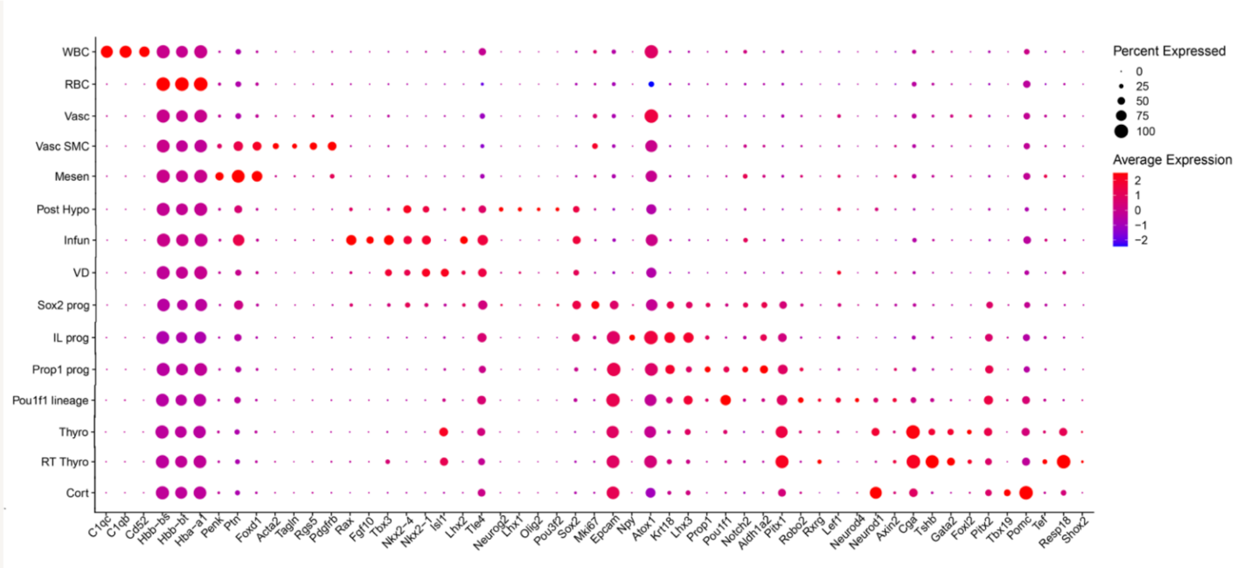

Supplemental Figure 3. Differences in gene expression associated with cell clusters.

A dot plot presents selected genes that are differentially expressed in cell clusters. Abbreviations: white blood cells (WBC), red blood cells (RBC), vasculature (Vasc), mesenchyme (Mesen), posterior hypothalamus (Post Hypo), infundibulum (Infun), ventral diencephalon (VD), progenitor (prog), intermediate lobe (IL), pars distalis thyrotrope (Thyro), rostral tip or pars tuberalis thyrotrope (RT Thyro), corticotrope (Cort).

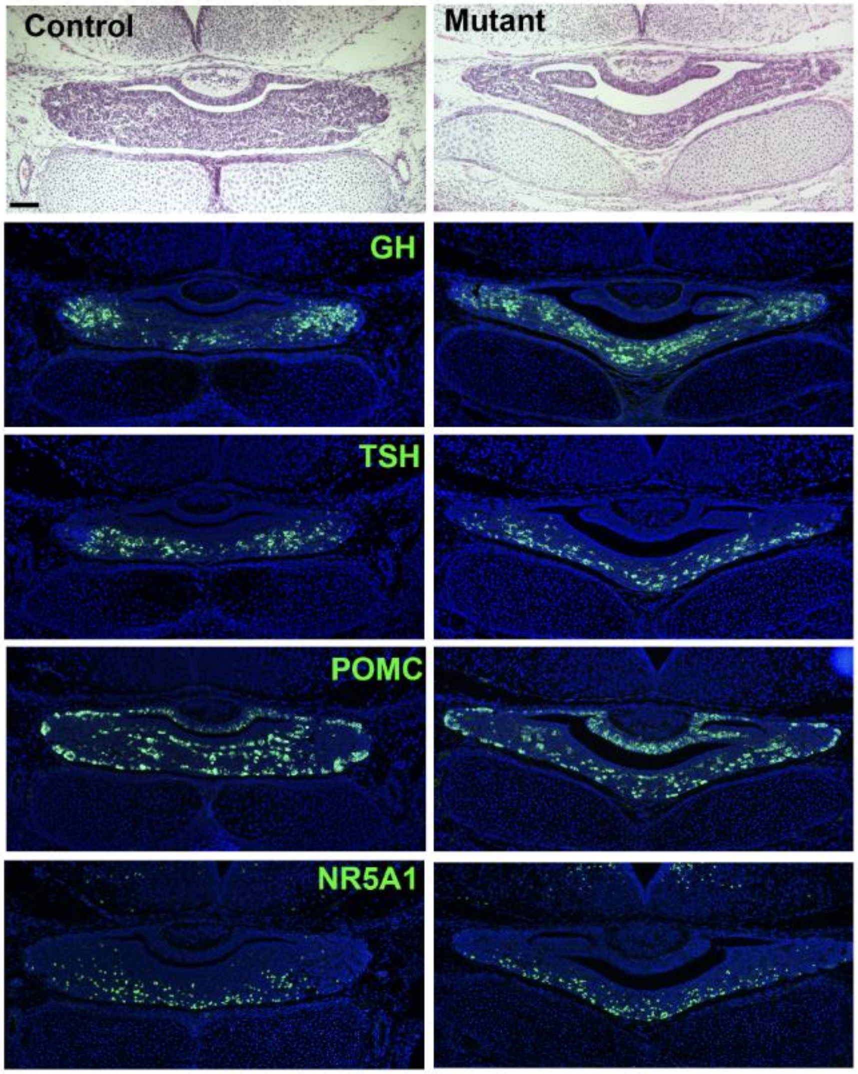

Supplemental Figure 4. Persistent dysmorphology and differentiation of gonadotrope and *Pou1f1* lineages in later development.

Pituitary glands were collected at e18.5 from controls and mutants and processed for histology by sectioning in the coronal plane. Hematoxylin and eosin staining reveals the dysmorphology that persists in the mutant pituitary. Immunostaining for NR5A1, GH, TSH and POMC did not reveal any obvious differences in mutants.

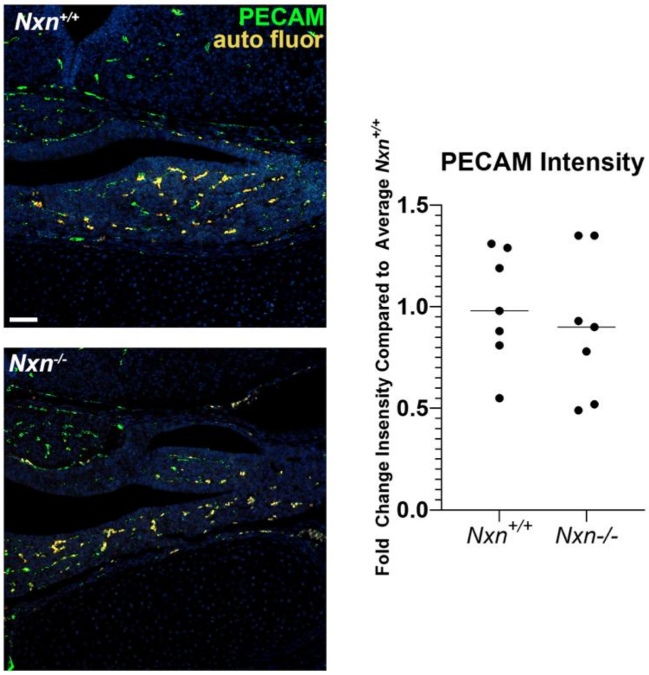

<u>Supplementary Figure 5. *Nxn* deficiency has no obvious effect on pituitary vasculature.</u>

A. To assess vascular development, pituitaries were collected from e18.5 wild type fetuses and *Nxn*$^{-/-}$ littermates, N=7/genotype. 2-5 coronal pituitary sections per individual were stained with PECAM1 antibodies (also known as CD31) and imaged by confocal microscopy at 20x with the green (Alexa488 laser) channel. Auto-fluorescent red blood cells were imaged with the red channel. Image J was used to quantify the signal intensity of the green and red channel images per area. PECAM signal intensity was calculated by subtracting the red signal from the green and averaged for the sections from a single individual. *Nxn* $^{-/-}$ values were normalized to the average wild type value.
